# Self-Reported Responses to Player Profile Questions Show Consistency with the Use of Complex Attentional Strategies by Expert Horseshoe Pitchers

**DOI:** 10.3389/fpsyg.2016.01028

**Published:** 2016-07-05

**Authors:** Jeffrey T. Fairbrother, Phillip G. Post, Sam J. Whalen

**Affiliations:** ^1^Department of Kinesiology, Recreation, and Sport Studies, University of TennesseeKnoxville, TN, USA; ^2^Department of Kinesiology and Dance, New Mexico State UniversityLas Cruces, NM, USA; ^3^SAIC USABirmingham, MI, USA

**Keywords:** attentional strategies, attention, mental skills, horseshoe pitching, expertise

## Abstract

The advantages on an external focus of attention have been demonstrated for a variety of sport tasks. The *constrained action hypothesis* (Wulf et al., 2001) argues that focusing externally on the movement effect results in the use of automated processes for movement control. In contrast, focusing internally in an attempt to control the movements of the body disrupts normally automated processes and degrades performance. Research on experts, however, suggests that they may adopt more complex attentional strategies. The present study provided a unique opportunity to examine expert horseshoe players’ attentional strategies as indicated by their self-reported responses to questions included in a National Horseshoe Pitchers Association (NHPA) player profile questionnaire. Responses submitted by 83 top NHPA players were examined to determine the frequency of references to the use of internal and external focus points and identify categories related to attentional strategies. Results indicated that the large majority of players reported using focus points that are consistent with an external focus of attention and that their thoughts corresponded to one or more categories related to technique, mental focus or concentration, general success, use of external focus cues, and emotional control. The findings are consistent with the view that experts may adopt complex attentional strategies that encompass both an external focus and thoughts about a variety of other performance related factors.

## Introduction

In order to optimize skill execution, athletes need to devote their attention to the most critical aspects of the performance environment and avoid possible distractions. Over the last several years, a growing body of research has demonstrated the benefits of adopting an external focus of attention when executing motor skills (Wulf, 2007, 2013). An external focus has been defined as one that directs the performer’s attention to the movement effect (e.g., a golfer focusing on the pendulum motion of the golf club or the trajectory of the ball). In contrast, an internal focus directs the performer’s attention to body mechanics (e.g., a golfer focusing on keeping the leading arm straight during a golf swing; Zachry et al., 2005; Wulf and Su, 2007). Previous research has shown that instruction and feedback that encourages learners/performers to adopt an external focus during skill execution improves performance outcomes, produces more efficient movements, and enhances skill acquisition to a greater extent than internal focus instructions or feedback (for a review see Wulf, 2007, 2013). The advantages of an external focus have been demonstrated for a variety of sport tasks, including basketball free throws (Zachry et al., 2005), volleyball serves (Wulf et al., 2002; Experiment 1), tennis ground strokes (Wulf et al., 2000; Experiment 1), golf pitching (Wulf et al., 1999), standing long jump (Porter et al., 2012), and soccer kicks (Wulf et al., 2003). By comparison, internal focus instructions and feedback have largely been shown to be less effective (Wulf, 2007).

The *constrained action hypothesis* has been forwarded to explain the advantages of an external focus (Wulf et al., 2001). According to this explanation, focusing on the movement effect results in the regulation of movements through automatic processes because it directs attention away from the conscious control of the movements themselves. Conversely, adopting an internal focus of attention constrains the motor system by disrupting the automatic processes that are normally associated with controlling movements effectively and efficiently (Vance et al., 2004; Zachry et al., 2005; Wulf, 2007). Support for the *constrained action hypothesis* comes from research examining how attentional focus instructions effect learners’ attentional capacity, muscular activation, and frequency of movement adjustments (Wulf, 2007, 2013). With respect to attentional capacity, Wulf et al. (2001) found that participants who adopted an external focus had significantly faster secondary probe reaction times compared to participants who used an internal focus while balancing on a stabilometer. The decreased secondary probe reaction times under the external focus condition were interpreted as an indication of more automated processing. With respect to muscular activation, Vance et al. (2004) found that participants who adopted an external focus demonstrated less EMG activity in both the agonist (i.e., bicep) and antagonist (i.e., triceps) muscles during a bicep curl compared to participants who used an internal focus. The results suggested that the external focus produced more efficient movements, consistent with those associated with automated behavior (e.g., reduced co-contraction of agonist and antagonist muscles or more efficient metabolic rate; Sparrow and Newell, 1998; Sparrow et al., 1999; Lay et al., 2002). In contrast, the internal focus introduced additional noise at a neuromuscular level, reflecting a relatively inefficient process associated with conscious control of the movement. With respect to postural adjustments, McNevin et al. (2003) found that participants who adopted an external focus demonstrated higher frequencies of postural adjustments than participants who used an internal focus. Such high frequency adjustments are thought to reflect the use of more rapid and automated reflex loops in postural control (e.g., Gurfinkel et al., 1995). Despite the empirical support for the *constrained action hypothesis*, other researchers have argued that external focus benefits are related to reduced attentional demands (Bell and Hardy, 2009) and might also depend upon the skill level of the performer (Perkins-Ceccato et al., 2003; Castaneda and Gray, 2007). Although these divergent views illustrate the complexity of attentional regulation during skilled performance, they do so in the context of a growing body of empirical evidence showing benefits of adopting an external focus.

Research examining attentional focus effects on experts has yielded intriguing results and suggests that it is time to broaden attentional focus research to explore how attention might be devoted to information not captured in the internal vs. external dichotomy. For example, Wulf (2008) examined balancing performance by expert acrobats and found that instructions to adopt *either* an external or internal focus of attention degraded performance compared to a control condition. Wulf (2008) argued that both types of instructions disrupted the acrobats’ use of automated processes to balance effectively. It is also possible that directing experts to focus on unfamiliar cues might negatively impact their performance. For example, Maurer and Munzert (2013; Experiment 1) found that skilled female basketball players had better free throw shooting performance when adopting a familiar attentional focus compared to when they adopted an unfamiliar one. The results showed no differences in free throw shooting performance between internal and external focus conditions. Performance differences were only observed between the familiar and unfamiliar attentional focus cue conditions. Maurer and Munzert (2013) suggested that any cue that directs performers to an unfamiliar focus can cause deautomization of a highly practiced skilled.

These prior results are consistent with the possibility that experts might use a more complex attentional strategy, which may include both internal and external foci. For example, in a survey of 58 professional dancers Guss-West and Wulf (2016) found that 36.1% of focused on internal cues, 27.7% on external cues, and 36.1% of the used a mix of internal and external focus cues while performing. Similarly, a majority (69%) of athletes on the US track and field team reported focusing on internal cues when competing (Porter et al., 2010). Presumably, an expert will have developed an effective attentional strategy that includes the most useful information needed to facilitate performance. In addition to using internal and external focus cues, experts might also allocate attention for other means (e.g., self-talk or arousal regulation strategies). Recently, Bernier et al. (2011) reported that expert golfers have relatively complex attentional focus patterns during training and competition. Their results indicated that the golfers directed attention toward several different aspects of performance, including technique, procedures, outcomes, psychological states (i.e., mental and emotional aspects of their performance), and various elements of the environment (some task relevant, some distractions). The observed patterns differed between training and competition. During training, the skilled golfers were more likely to focus on the movement and resulting kinesthetic sensations. During competition, the golfers were more likely to attend to movement outcomes, psychological states (e.g., focus or confidence), and visual information (e.g., hand placement or the target). Bernier et al.’s (2011) results suggest that experts may focus on several aspects of their performances beyond internal and external focus cues.

Interestingly, research demonstrating that attentional demands fluctuate during skill execution (Castiello and Umilta, 1988; Prezuhy and Etnier, 2001) suggests that experienced performers have the opportunity to adopt complex patterns of attentional focus, at least in closed skills such as golfing or horseshoes. For example, Prezuhy and Etnier (2001) found that skilled horseshoe players had significantly faster secondary probe reaction times at the end-point of their backswing compared to at the beginning and ending of their movement. Similar fluctuations in attention have also been found during skilled performer’s execution of volleyball sets (Sibley and Etnier, 2004) and volleyball service receiving (Castiello and Umilta, 1988). These results indicate that at certain points during the execution of a motor skill, experts will have some portion of their attentional capacity free to focus on more than one source of information. In horseshoes for example, attentional resources during the backswing might be directed toward a combination of foci related to the feel of the swing, the stake, and general performance self-talk (e.g., “get a ringer”).

The literature in applied sport psychology also suggests that experts attend to a wide range of information related to the technical (e.g., technical reminders), tactical (e.g., strategy), mental (e.g., confidence, focus, self-talk, and pre-performance routines), and emotional (e.g., staying relaxed, self-control, arousal regulation, and stress management) aspects of their sport performance (Ravizza and Osborne, 1991; Landin and Hebert, 1999; Wrisberg, 2007; Williams, 2010). Interviews with Canadian Olympians have revealed that the skills associated with maintaining concentration and avoiding distractions (e.g., routines, self-talk, etc.) are key components of success (Orlick and Partington, 1988). More recently, Post and Wrisberg (2012) found that highly skilled gymnasts used snapshot imagery during competition routines. Together, these findings indicate that experts devote substantial attention to technical, tactical, mental, and emotional aspects of their performance, and that they have the attentional capacity to concurrently attend to more than one source of information.

Currently, how experts allocate attention to different sources of information during their performance is not well understood. Therefore, further investigation is needed to describe the nature of experts’ attentional processes during performance. Such investigations will enable researchers to understand demands on information processing and strategies employed by experts to manage limited attentional resources. A better understanding of these strategies may assist movement practitioners working with performers to enhance skill acquisition and motor performance. Therefore, the purpose of the present study was to supplement previous research by examining self-reported focus points and thoughts published in the player profiles of expert horseshoe players. The present study provided a unique opportunity to examine how experts expressed their thoughts about performance when answering questions that presumably have some bearing on potential attentional strategies used during horseshoe pitching. Horseshoe pitching is an ideal sport to examine experts’ attentional strategies given that the player can take up to 30 seconds to complete the two required pitches in an inning. Similar to golf, the relatively slow pace allows time for an extended pre-performance routine. Moreover, the preparatory phases (stance, sighting, and backswing) are long enough to allow for attentional shifting. This latter point is supported by Prezuhy and Etnier’s (2001) results that showed secondary task performance varied depending upon when a reaction time probe was delivered during n pitching. Examining how horseshoe pitchers manage their attention will contribute to a better understanding of the potential complexity of attentional strategies adopted by expert performers.

National Horseshoe Pitchers Association (NHPA, 2016) rules and regulations indicate that horseshoe pitching requires players to pitch a horseshoe 37 ft (men) or 27 ft (women) to a stake at least 14 in but not more than 15 in above the level of the pitching platform. Horseshoe pitching is divided into innings. Each inning consists of four pitched shoes, two by each contestant. Contestants have 30 s to deliver both shoes. After all shoes in an inning have been pitched, the thrown horseshoes are considered live (i.e., playable shoes that rest in the pit area no more than 6 in from the stake) or dead (i.e., non-playable shoes – a foul, a canceled-out ringer, or a shoe outside of 6 in) and are scored accordingly (i.e., 1 pt for a live shoe or 3 pt for a ringer). The pitcher who first reaches 40 pt is deemed the winner.

The responses examined in the present study were from the top men and women in the NHPA. A secondary data analysis approach was used to examine self-reported responses to two questions in previously published player profiles. Responses to these questions were examined through the lens of existing research on attentional strategies in in the motor learning and sport psychology literature. Specifically, responses to both questions were examined to identify statements consistent with internal and external foci as described in Wulf’s (2007) classification of attentional focus. Responses were also examined to identify categories that were consistent with a variety of attentional strategies identified in the motor learning and sport psychology literature (e.g., an outcome focus on technique or the use of arousal regulation skills).

## Materials and Methods

### Data Selection

The Institutional Review Board (IRB) at the University of Tennessee approved the study. After receiving IRB approval data were retrieved from player profiles and published on the NHPA website^1^. Completed player profiles (*N* = 83) and corresponding statistics were selected based on published rankings to include the top men (*n* = 43) and women (*n* = 40) with profiles. Level of expertise was determined by NHPA national statistics (NATSTATS) on ringer percentages. NATSTATS listed 3,116 women with average ringer percentage that ranged from 0.5 to 85.22%. Ringer percentage is the average of the top three games for the year. Individual game percentages were calculated by dividing total number of ringers by total throws in the game (Goodrich, 2007). Women in our data set had an average ringer percentage from 44.29 to 85.22%. Thus, our 40 women came from the Top 240 out of 3,116 performers (7.70%). NATSTATS listed 7,377 men with average ringer percentage ranging from 0.25 to 87.87%. Men in our data set had an average ringer percentage from 55.22 to 87.87%. Thus, the 43 men included in the current analysis were from the top 181 out of 7,377 performers (2.45%).

### Analyses

All three authors were involved in the data analyses. The first and third authors worked together to code player responses and the second author worked independently. Two profile questions were analyzed for the purposes of the present study: *What is your focus point when pitching?*; and, *Any particular thoughts while pitching?* Statements within the open-ended responses to the first question were coded as external, internal, or other. Coding decisions were based on Wulf’s (2007) conceptualization of attentional focus. Statements were considered to have an external focus if they included reference to any point outside a person’s body or the movement effect (i.e., manipulating the horseshoe). Statements were considered to have an internal focus if they included reference to the movement pattern or technique (e.g., focusing on the arm movement). The *other* category was used to classify statements that did not fall into either of the previous two categories. After the initial coding of player statements the raters discussed any discrepancies in coding until all individuals agreed upon the most accurate classification (initial discrepancies represented 0.21% of all 480 coding decisions). A chi-square procedure with an alpha level of 0.05 was then used to analyze the total number of external, internal, and other responses.

The open-ended responses to the second item (*Any particular thoughts while pitching?*) were examined using procedures developed by Thomas and Pollio (2002). These procedures involve at least two researchers reading the text several times aloud together. After reading each response, the researchers discuss the statement and identified segments containing meaningful information. The group then works together to come up with higher-order themes that represent identified meaning units within the text. A third researcher then acts as reliability check and reads the text independently. This ensures that identified meaning units and themes are representative of the data (i.e., text). Consistent with these procedures, teach player’s response was read several times aloud by the first and third author. After reading each response, the first and third author discussed the statement and identified segments containing meaningful information. Information-rich statements were then identified as *meaning units*. After reading all of the participants’ statements, the authors compared and regrouped the meaning units into distinct categories that represented the players’ thoughts while pitching. Categories that emerged were labeled according to the common features shared by the meaning units within each category. The second author then independently examined the player’s statements using the same procedures, first identifying meaning units and then regrouping them into distinct categories. After the initial analysis, the three authors discussed any discrepancies until agreement was reached (initial discrepancies represented 1.90% of all 948 meaning units and category identification decisions). A chi-square procedure with an alpha level of 0.05 was then used to analyze the total number of responses in each of the categories.

## Results

### What is Your Focus When Pitching?

**Table 1** shows the frequencies of players reporting each focus category identified in the responses. Three players did not respond to the question and four provided information that fit more than one of the three coding categories. For the latter cases, each instance of each coding category was counted. A majority of players (96%) gave responses that included statements containing an external focus point while only small number (6%) included internal focus points. The chi-square analysis revealed that focus points were not equally distributed in the sample, χ^2^_0.05 (2)_ = 125.46, *p* < 0.001. Specifically, responses included a higher than expected frequency of external focus points. The majority of players reporting an external focus point described directing their attention to the stake. Examples of responses included, “I focus on the entire stake,” “half way up the stake,” “the top of the stake,” and “I focus about six inches from the bottom of the stake.”

**Table 1:** Frequency and percentage of players for each category of responses to, “What is your focus point when pitching?”

	Frequency (*n)*	%
External	77	96.25
Internal (process)	5	6.25
Other	2	3.75

*N = 80 player profiles (41 = men’s division, 39 = women’s division); Percentages do not sum to 100% because some player responses produced meaning units for more than one category. Four of the five players who produced meaning units in the internal category also had a meaning unit in the external category. Both players who produced meaning units in the other category did not produce meaning units in the other two categories*.

### Any Particular Thoughts While Pitching?

**Table 2** includes the categories that emerged and the number of meaning units within each category. A majority of responses (88%) reported only one or two thoughts. Five players did not respond to the question, 49 produced only one meaning unit, 20 produced two, eight produced three, and one produced four. The analysis of the data revealed a total of 117 meaning units. Meaning units were organized into the following six categories: *thoughts about technique*; *mental focus/concentration*; *thoughts about general success*; *use of external focus cues*; *emotional control*; or *other*. The number of meaning units within each category varied. Despite the fact that certain categories did not contain a high number of meaning units, their inclusion was necessary to provide a complete depiction of the variety of thoughts reported. In the following sections, each category is discussed and representative meaning units are provided. The categories are presented from the highest to lowest reported frequency. The chi-square analysis revealed that thoughts were not equally distributed in the sample, χ^2^_0.05 (5)_ = 46.74, *p* < 0.001. Specifically, responses included higher than expected frequencies for *thoughts about technique*.

**Table 2:** Frequency and percentage of players who produced meaning units for each category related to, Any particular thoughts while pitching?

Category	Frequency (*n)*	%
Thoughts about technique	45	57.69
Mental focus/concentration	21	26.92
Thoughts about general success	19	24.36
Use of external focus cues	14	17.95
Emotional control	11	14.10
Other	7	8.97

*N = 78 player profiles (39 = men’s division, 39 = women’s division); Percentages do not sum to 100% because some player responses included meaning units corresponding to multiple categories*.

#### Thoughts about Technique

This category represented the majority of meaning units. Process thoughts were reflected in statements that referred to thoughts about the execution of movements. Specifically, the players reported thoughts about task-oriented aspects of the horseshoe pitch. For example, players described thinking about how to execute the pitch correctly (e.g., easy release, good timing, or smooth weight shift). One player responded, “I think about keeping my shoulders square, keeping my body level when moving toward the stake, and holding the follow through. A good and simple form is important for me”. Other representative meaning units of process focus include “Just telling myself to follow through,” “Shoulders square head level throughout the delivery and a smooth follow through,” and “Keeping it slow and fluid and watch that backswing and follow thru.”

#### Mental Focus/Concentration

The mental focus/concentration category included statements about players concentrating fully on the performance and avoiding distraction. One player’s response included the statement, “Focus!! I try to stay in the ZONE.” Other representative meaning units included “I try to block everything out,” “I concentrate only on the next pitch,” and “concentrate.”

#### Thoughts about General Success

General success statements referred to generic thoughts about success or abstract representations of performance (e.g., making a ringer or trying to win). All of these statements were positive thoughts about performance. For example, one player indicated that he thought about “Getting a double and beating my previous ringer percentage.” Other representative meaning units of general success included “Just throw ringers,” “Throw the shoe out there and get lots of ringers,” and “I focus on the win.”

#### Thoughts about External Focus Cues

This category included statements about thoughts directed toward objects (e.g., the stake or the shoe). Several of these statements were similar to the responses to the first question that indicated an external focus point. For instance one player described thinking about “Banging the stake.” Other representative meaning units of external thoughts included “Watch the shoe hit the peg,” “focus on the stake,” and “hitting the peg.”

#### Emotional Control

Emotional control statements referred to the use of emotional regulation strategies (e.g., staying calm or relaxed). Players indicated that it was important to slow down and relax while performing. One player reported thinking, “Take a deep breath and relax.” Other representative meaning units of emotional control included “Stay relaxed,” “I always try to slow down and relax,” “stay calm,” and “relax.”

#### Other

The other category included statements that did not fall into any of the previous categories. These statements appeared to have no connection to a player’s performance. For example one player described thinking “Can’t wait to the game is over, so I can talk to other pitchers.” Other representative meaning units for this category included “I sing,” “Never watch the shoe,” and “counting 8 or 9 out of 10.”

## Discussion

The majority of previous research has shown performance advantages of adopting an external focus of attention for learners and mid-level performers (Wulf, 2007). However, previous attentional focus research with experts has shown inconsistent results. Research with skilled golfers (i.e., handicaps under ten) suggests that external focus instructions facilitate performance (Wulf and Su, 2007; Bell and Hardy, 2009), while research with acrobats suggests that any type of instructional focus disrupts automated control processing (Wulf, 2008). Furthermore, prior motor control research demonstrates that skilled performers’ attention fluctuates during skill execution (Prezuhy and Etnier, 2001; Sibley and Etnier, 2004). Taken together, these findings suggest it is possible that experts may adopt complex attentional strategies that include both internal and external foci, so that any instruction to exclusively use either one deprives experts of information that they would normally use during performance. The purpose of the present study was to supplement previous research by examining self-reported focus points and thoughts published in the player profiles of expert horseshoe players.

An important result of the present study was the significant difference found for expected frequencies of statements in response to the question about focus points. A large number of players (96%) reported using a focus point consistent with an external focus of attention as opposed to an internal focus of attention (6%). A review of these comments indicated that a number of these players focused on specific parts (e.g., top, bottom, or middle) of the stake during their pitch. These results were likely related to the fact that horseshoe pitching is a visually guided targeting task. Although some players reported focusing internally, it is possible that others interpreted the question as asking about visual focus. Nevertheless, it is plausible that visual attention is closely related to attentional focus in targeting tasks such as horseshoe pitching, especially in those who regularly achieve objective success in terms of accuracy. A dissociation between mental processes and visual attention – while possible – would likely be maladaptive. For less skilled players, this might represent a barrier to achieving high performance levels and future research should be directed toward addressing this issue. The responses did not suggest that participants shifted either their visual or attentional focus during the pitch. The findings suggest that a majority of expert horseshoe players adopt a focus strategy that is consistent with using an external focus of attention while pitching. To the extent that there is no dissociation between visual attention and attentional focus, the results are were consistent with previous research showing an external focus benefit for expert golfers (Wulf and Su, 2007; Bell and Hardy, 2009).

An additional aim of the present study was to evaluate the player’s self-reported thoughts while pitching. The examination of the open-ended question “Any particular thoughts while pitching” indicated a variety of responses. The majority of meaning units (66%) suggested that players thought about thought about the technical, mental (i.e., focus), and/or emotional aspects of their performance. These meaning units emerged from 60 of the 78 total responses. The technique category referred to thoughts about proper pitching execution or set-up (38% of all meaning units). For example, some players reported thinking about shifting their weight or following through while pitching. These types of responses suggest that, at some point during their performance, players directed attention to internal mechanics (i.e., body mechanics) associated with pitching. However, it is uncertain at what time (e.g., right before, during, or after) players devoted attention to the process of their performance. Responses also indicated that 18% of all meaning units referred to thoughts directed toward mental focus/concentration and 9% referred to thoughts about emotional control. Together, these results indicated that 18 players (23% of all players) directed attention toward mental and/or emotional aspects of their performance. Specifically, some players thought about maintaining concentration or staying relaxed during their performance.

These findings are consistent with literature in sport psychology that suggests experts also direct attention to technical, tactical, mental, and/or emotional aspects of their performance (Ravizza and Osborne, 1991; Landin and Hebert, 1999; Wrisberg, 2007; Williams, 2010; Bernier et al., 2011). Specifically, research with experts has indicated that these athletes use strategies that can include process cues, mental skills (i.e., self-talk, imagery, etc.), pre-performance routines, or emotional regulation techniques to avoid distractions (Orlick and Partington, 1988; Ravizza and Osborne, 1991). The findings of the present study suggest that expert horseshoe players might adopt similar strategies to facilitate performance. The focus points and thoughts reported by the expert horseshoe players in the current study included content beyond the internal and external focus dichotomy, illustrating that they may be able to maintain a high level of performance using attentional strategies that also include thoughts about other aspects of performance (i.e., technique reminders, mental focus, and emotional control). One possibility is that expert performers in the current study use strategies consistent with Singer’s (1986) five-step mental approach to assist performance and learning for self-paced motor tasks (Singer and Cauraugh, 1985). Specifically, the five-step approach includes sequential phases for *Readying*, *Imaging*, *Focusing*, *Executing*, and *Evaluating*. According to this model, a task such as horseshoe pitching would allow performers to spend time during the Readying, Imaging, and Focusing phases directing attention to aspects of performance that help them prepare for the throw (i.e., technique reminders, mental focus, and emotional control). Just prior and during the Execution phase, players could then shift their attention to an external focus (e.g., the stake). Presumably, the Evaluation phase might include watching the flight of the shoe and where it comes to rest as well as monitoring other inherent feedback from the task. Further research will be needed to determine how well the model fits actual horseshoe pitching, but it offers a plausible sequence that is consistent with the evidence produced by the current study.

Overall the present investigation suggests that expert horseshoe players primarily adopt an external focus point during the moment of skill execution, but that at some point during the time course of their performances, they also direct attention inwardly as shown by the meaning units falling into the technical, cognitive, and/or emotional categories. The present findings should be interpreted with some caution, however, because it is uncertain how players interpreted the questions “What is your focus when pitching?” and “Any particular thoughts while pitching”. Although the results aligned well with previous attentional focus literature it is possible that some players interpreted the *focus point* question as referring to visual focus. Additionally, the responses to the *thoughts* question might have included thoughts that occurred outside the act of pitching itself (e.g., general thoughts occurring during an entire match) and might have represented only a portion of the players’ thoughts (i.e., some might have provided *sample thoughts*). Nevertheless, the results suggest that expert horseshoe pitchers behave in ways that are consistent with effective attentional strategies emerging from the motor learning and sport psychology literature.

Despite these limitations, the results of the present study suggest that expert horseshoe players’ attentional strategies may be more complex than what has been previously described. However, future empirical research is needed to examine experts’ attentional strategies during performance. Specifically, future research might include qualitative interviewing to explore experts’ attention over the time course of their performances or the use of verbal protocol analysis (Ericsson and Simon, 1993) to document what experts are thinking as the pitching action unfolds. Such investigations with a variety of athletes from different skill levels would shed light on the characteristic ways that expert performers manage attention during skill execution.

### Implications

The present study has implications for both movement practitioners and sport psychology consultants. An important part of skill instruction is directing performer’s attention to the most relevant aspects of the task. It appears that in most cases movement practitioners should direct performer’s attention to the movement effect (i.e., an external focus). It may be that an external focus instruction works so well because it focuses performers on what they need to do to facilitate performance during the execution phase of the task, particularly for tasks that have a goal of accurately projecting an object toward a target (i.e., horseshoe pitching, basketball shooting, etc.). Presumably, thoughts prior to execution that are directed toward technical, tactical, mental, or emotional aspects might not be detrimental unless carried into the execution phase. Further research will be needed to determine the extent to which this is true. For sport psychology consultants, the results imply that it is important for experts to devote attention to the technical, mental, and emotional aspects of their performance, however, it may not be advantageous to focus on these aspects during the execution of the skill. Therefore, mental skills training may be effective because it allows performers to self-regulate thoughts between performance attempts, thus minimizing external distractions and allowing performers to refocus on the task at hand just prior to execution (i.e., process cue reminders). Given the robust findings related to external focus benefits, however, it is important for consultants to teach athletes how to optimize transitions from internal regulation or reminders to an external focus when executing a skill.

## Author Contributions

All authors contributed to collecting and analyzing the data. All of the authors also contributed to writing the manuscript. JF was the project lead.

## Conflict of Interest Statement

The authors declare that the research was conducted in the absence of any commercial or financial relationships that could be construed as a potential conflict of interest.

## Footnotes

1 http://www.horseshoepitching.com
